# The association between prothrombin time-international normalized ratio and long-term mortality in patients with coronary artery disease: a large cohort retrospective study with 44,662 patients

**DOI:** 10.1186/s12872-022-02619-4

**Published:** 2022-06-29

**Authors:** Liwei Liu, Ming Ying, Shiqun Chen, Qiang Li, Guanzhong Chen, Huanqiang Li, Ziling Mai, Yibo He, Bo Wang, Danyuan Xu, Zhidong Huang, Xiaoming Yan, Ning Tan, Zhujun Chen, Jin Liu, Yong Liu

**Affiliations:** 1 Department of Cardiology, Guangdong Provincial Key Laboratory of Coronary Heart Disease Prevention, Guangdong Cardiovascular Institute, Guangdong Provincial People’s Hospital, Guangdong Academy of Medical Sciences, South China University of Technology, Guangzhou, 510080 China; 2 Present Address: Department of Cardiology, Zhongshan Hospital, Fudan University, Shanghai Institute of Cardiovascular Diseases, Shanghai, China; 3 School of Medicine, Guangdong Provincial People’s Hospital, South China University of Technology, Guangzhou, 510100 China; 4 Department of Information Technology, Guangdong Provincial People’s Hospital, Guangdong Academy of Medical Sciences, Guangzhou, 510080 China

**Keywords:** Coronary artery disease, International normalized-ratio, All-cause mortality

## Abstract

**Background:**

The association between prothrombin time-international normalized ratio (PT-INR) and long-term prognosis among patients with coronary artery disease (CAD) without atrial fibrillation or anticoagulant therapy was still unclear. We analyzed the association of PT-INR levels and long-term mortality in a large cohort of CAD patients without atrial fibrillation or using of anticoagulant drugs.

**Methods:**

We obtained data from 44,662 patients who were diagnosed with CAD and had follow-up information from January 2008 to December 2018. The patients were divided into 4 groups (Quartile 1: PT-INR ≤ 0.96; Quartile2: 0.96 < PT-INR ≤ 1.01; Quartile3: 1.01 < PT-INR ≤ 1.06; Quartile4: PT-INR > 1.06). The main endpoint was long-term all-cause death. Kaplan–Meier curve analysis and Cox proportional hazards models were used to investigate the association between quartiles of PT-INR levels and long-term all-cause mortality.

**Results:**

During a median follow-up of 5.25 years, 5613 (12.57%) patients died. We observed a non-linear shaped association between PT-INR levels and long-term all-cause mortality. Patients in high PT-INR level (Quartile4: PT-INR > 1.06) showed a significantly higher long-term mortality than other groups (Quartile2 or 3 or 4), (Compared with Quartile 1, Quartile 2 [0.96 < PT-INR ≤ 1.01], aHR = 1.00, 95% CI 0.91–1.00, *P* = 0.99; Quartile 3 [1.01 < PT-INR ≤ 1.06], aHR = 1.10, 95% CI 1.01–1.20, *P* = 0.03; Quartile 4 [PT-INR > 1.06], aHR = 1.33, 95% CI 1.22–1.45, *P* < 0.05).

**Conclusions:**

Our study demonstrates high levels of PT-INR were associated with an increased risk of all-cause mortality.

**Supplementary Information:**

The online version contains supplementary material available at 10.1186/s12872-022-02619-4.

## Background

The prothrombin time-international normalized ratio (PT-INR), as a reliable marker of coagulation abnormalities [1], usually used for monitoring patients on oral anticoagulant therapy. A lower PT-INR level reflects a higher risk of thrombosis [2], while a higher PT-INR level indicates a higher risk of bleeding [3].

Previous studies have focus on the optimal range of PT-INR in patients treating with oral anticoagulant therapy, such as atrial fibrillation (AF) [4, 5], in which has found the significant relationship between PT-INR level and long-term mortality [6, 7]. PT-INR is regulated by multiple coagulation factors synthesized in the liver and is widely used to monitor anticoagulation, to assess hepatic function, and to evaluate coagulation abnormalities. Previous studies revealed that PT-INR level was closely related to status of inflammation among serious of diseases [8, 9]. Similarly, some studies showed a higher PT-INR level was significantly associated mortality in patients undergoing endarterectomy [10], or with acute heart failure [11]. After excluding patients on oral anticoagulation therapy or AF, PT-INR still become an independent prognostic factor among ischemic stroke patients [12]. Coronary heart disease is the most common type of organ disease caused by atherosclerosis, and it is often accompanied by a high risk of thrombosis and bleeding. Most studies on PT-INR were mostly limited to patients receiving anticoagulation therapy. However, the relationship between PT-INR and long-term all-cause mortality in CAD patients without atrial fibrillation is not as clearly defined, particularly in the absence of anticoagulant therapy.

Therefore, we aim to investigate the association between the PT-INR and all-cause mortality in CAD patients without atrial fibrillation or oral anticoagulant therapy.

## Methods

### Study design and participants

This observational prospective study was completed in Guangdong Provincial People's Hospital (ClinicalTrials.gov NCT04407936) [13]. A total of 88,939 patients underwent coronary angiography from January 2008 to December 2018. 59,667 patients with a final diagnosis of CAD according to the 10th Revision Codes of the International Classification of Diseases (ICD-10; I20.xx–I25.xx, I50.00001 and I91.40001, Additional file 1: Table S1). A total of 44,662 CAD patients were included in the final analysis after excluding patients who lacked off PT-INR levels, follow-up information and diagnosed with AF, took warfarin or new oral anticoagulant (Additional file 3: Figure S1). This research program was performed according to the Declaration of Helsinki and approved by The Ethics Committee of Guangdong Provincial People's Hospital.

Baseline information such as demographic characteristics, clinical settings, laboratory examinations and medications at discharge were extracted from the electronic Clinical Management System of the Guangdong Provincial People’s Hospital.

PT-INR was measured using the reagent, HemosIL RecombiPlasTin 2G on a STA-R EVOLUTION automated coagulation analyzer (Diagnostica Stago, Asnières, France). Coronary angiography (CAG) or percutaneous coronary intervention (PCI) was performed in accordance with standard clinical practice guidelines [14, 15]. Additional informed consent was obtained from all patients for whom identifying information is included in this article.

### Definition and endpoint

We calculated the estimated glomerular filtration rate (eGFR) by applying the Modification of Diet in Renal Disease (MDRD) equation [16], chronic kidney disease (CKD) was defined an estimated glomerular filtration rate (eGFR) ≤ 60 mL/min/1.73 m^2^. Congestive heart failure (CHF) was defined as New York Heart Association (NYHA) class > 2 or Killip class > 1. Diabetes mellitus (DM) and hypertension were defined using the ICD-10 code (Additional file 1: Table S1). The primary endpoint of this study was long-term all-cause death, incident events were defined as the first event occurring between the date of enrollment and the end of follow-up of December 31, 2018. Trained nurses monitored and recorded follow-up data through outpatient interviews and telephones.

### Statistical analysis

Statistical analysis for this study was performed from January 1, 2008 to December 31, 2018. We divided the patients into 4 groups according to the quartile of PT-INR level (Quartile 1: PT-INR ≤ 0.96; Quartile2: 0.96 < PT-INR ≤ 1.01; Quartile3: 1.01 < PT-INR ≤ 1.06; Quartile4: PT-INR > 1.06). We reported descriptive statistics by means (SD), median (interquartile range [IQR]), or number and percentage when appropriate. The differences between different groups were analyzed with one-way analysis of variance (ANOVA). When analyzing categorical data, we used the Pearson chi-squared test. Kaplan–Meier methods and survival curves were used for prognosis analysis. Log-rank test was conducted to compare the survival differences between the four groups of patients.

We used cox proportional hazards regression models and restricted cubic splines to evaluate the relationship between PT-INR levels and all-cause mortality in CAD patients. Hazard ratios and 95% CIs are reported. Model 1 was unadjusted, Model 2 adjusted for age (as continuous variable) and gender, Model 3 was adjusted for CKD. Model 4, as the primary results, was adjusted with the variables which were significant at *P* < 0.05 according to univariate Cox proportional hazards regression, and associated with mortality according to clinical experience (included history of present illness information). The proportional hazards assumption was tested with the use of Schoenfeld residuals. We conducted sensitivity analysis in different subgroups stratified by PCI, AMI, CKD and DM. All data analyses were performed using R (version 3.6.3; R Core Team, Vienna, Austria). *P* values < 0.05 were considered to represent statistical significance.

## Result

### Clinical characteristics

A total of 44,662 patients were included in the final analyses. Baseline clinical of the study patients are shown in Table 1. The mean age was 62.96 ± 10.71 years, and 33,938 (75.99%) was male. The distribution of PT-INR as follows: mean, 1.02 ± 0.13, and patients were divided into four groups: (Quartile 1: PT-INR ≤ 0.96; Quartile 2: 0.96 < PT-INR ≤ 1.01; Quartile 3: 1.01 < PT-INR ≤ 1.06; Quartile 4: PT-INR > 1.06). 8544 (19.15%) patients with acute myocardial infarction (AMI), 7833 (21.56%) patients complicated with CKD and 3830 (8.58%) patients identified in CHF, 33,284 (74.52%) patients underwent PCI treatment (Table 1).

**Table 1:** Baseline characteristics of the patients

Characteristic	PT-INR level quartile					
	Overall	≤ 0.96	0.96–1.01	1.01–1.06	> 1.06	*p* Value
	(n = 44,662)	(n = 12,014)	(n = 12,389)	(n = 9936)	(n = 10,323)	
*Demographic*						
Age, year	62.96 (10.71)	60.98 (10.15)	62.31 (10.54)	63.75 (10.72)	65.28 (11.01)	< 0.001
Male, n (%)	33,938 (75.99)	8583 (71.44)	9353 (75.49)	7688 (77.38)	8314 (80.54)	< 0.001
*Medical history*						
AMI, n (%)	8544 (19.15)	1447 (12.06)	1931 (15.60)	1973 (19.88)	3193 (30.98)	< 0.001
CHF, n (%)	3830 (8.58)	601 (5.01)	761 (6.15)	820 (8.26)	1648 (15.98)	< 0.001
Hypertension, n (%)	25,415 (56.98)	6920 (57.68)	7021 (56.74)	5732 (57.76)	5742 (55.70)	0.007
DM, n (%)	12,423 (27.85)	3582 (29.85)	3384 (27.35)	2564 (25.84)	2893 (28.07)	< 0.001
PCI, n (%)	33,284 (74.52)	8746 (72.80)	9142 (73.79)	7464 (75.12)	7932 (76.84)	< 0.001
CKD, n (%)	7833 (21.56)	1479 (15.49)	1803 (18.18)	1764 (21.77)	2787 (31.79)	< 0.001
*Laboratory test*						
INR	1.02 (0.13)	0.93 (0.03)	0.99 (0.01)	1.04 (0.01)	1.15 (0.20)	< 0.001
D-dimer (ng/mL)	624.48 (1256.03)	474.66 (810.95)	514.92 (863.34)	605.73 (1116.67)	949.80 (1956.06)	< 0.001
FIB, g/L	4.12 (1.28)	4.03 (1.04)	4.07 (1.18)	4.14 (1.31)	4.26 (1.59)	< 0.001
ALT (U/L)	34.36 (84.89)	31.91 (28.61)	30.99 (54.32)	30.65 (31.11)	44.78 (159.84)	< 0.001
AST (U/L)	48.92 (196.68)	33.70 (44.46)	36.43 (55.56)	42.20 (70.42)	88.06 (393.12)	< 0.001
WBC, 10^9^/L	7.97 (2.71)	7.73 (2.14)	7.73 (2.31)	7.84 (2.60)	8.67 (3.59)	< 0.001
HGB, g/L	133.13 (16.75)	136.64 (14.81)	134.83 (15.58)	132.29 (16.44)	127.82 (18.98)	< 0.001
CHOL, mmol/L	4.54 (1.21)	4.87 (1.27)	4.59 (1.18)	4.41 (1.13)	4.21 (1.14)	< 0.001
TRIG, mmol/L	1.67 (1.22)	2.05 (1.68)	1.69 (1.11)	1.53 (0.93)	1.34 (0.76)	< 0.001
LDLC, mmol/L	2.80 (0.97)	2.97 (1.01)	2.84 (0.97)	2.73 (0.93)	2.63 (0.94)	< 0.001
HDLC, mmol/L	1.00 (0.26)	1.04 (0.27)	1.00 (0.25)	0.98 (0.25)	0.95 (0.25)	< 0.001
HbA1c, %	6.56 (1.42)	6.69 (1.52)	6.53 (1.38)	6.51 (1.39)	6.50 (1.37)	< 0.001
URIC, μmol/L	393.52 (110.49)	393.45 (102.21)	389.57 (102.89)	390.74 (109.23)	401.10 (128.07)	< 0.001
eGFR, ml/min/1.73 m^2^	77.95 (25.22)	82.87 (25.19)	79.64 (23.69)	77.33 (24.39)	71.25 (26.18)	< 0.001
ALB, g/L	36.37 (4.22)	37.40 (3.79)	36.93 (3.88)	36.31 (4.03)	34.55 (4.65)	< 0.001
Hs-CRP, mg/L	12.07(22.75)	6.24 (11.82)	8.66 (18.00)	11.60 (23.36)	23.40 (40.60)	< 0.001
*Medication*						
Antiplatelet, n (%)	42,965 (97.41)	11,606 (97.34)	11,945 (97.24)	9596 (97.59)	9818 (97.54)	0.318
ACEI/ARB, n (%)	22,110 (50.13)	5801 (48.65)	6130 (49.90)	4942 (50.26)	5237 (52.03)	< 0.001
Beta-blockers, n (%)	35,957 (81.52)	9804 (82.23)	10,039 (81.72)	8008 (81.44)	8106 (80.53)	0.012
Statin, n (%)	42,274 (95.85)	11,488 (96.35)	11,812 (96.16)	9434 (95.94)	9540 (94.77)	< 0.001
Death, n (%)	5613 (12.57)	1116 (9.29)	1426 (11.51)	1249 (12.57)	1822 (17.65)	< 0.001

*AMI* acute myocardial infarction, *CHF* congestive heart failure, *DM* diabetes mellitus, *PCI* percutaneous coronary intervention, *CKD* chronic kidney disease, *WBC* white blood cell, *HGB* hemoglobin, *CHOL* serum total cholesterol, *LDL-C* low-density lipoprotein cholesterol, *HDL-C* hight-density lipoprotein cholesterol, *HbA1c* Glycated hemoglobin, *eGFR* estimated glomerular filtration rate, *ALB* albumin, *Hs-CRP* hypersensitive C-reactive protein, *ACEI/ARB* angiotensin-converting enzyme inhibitor/angiotensin receptor blocker, *AST* aspartate transaminase, *ALT* alanine aminotransferase, *FIB* fibrinogen

### Main outcomes

**Fig. 1:**
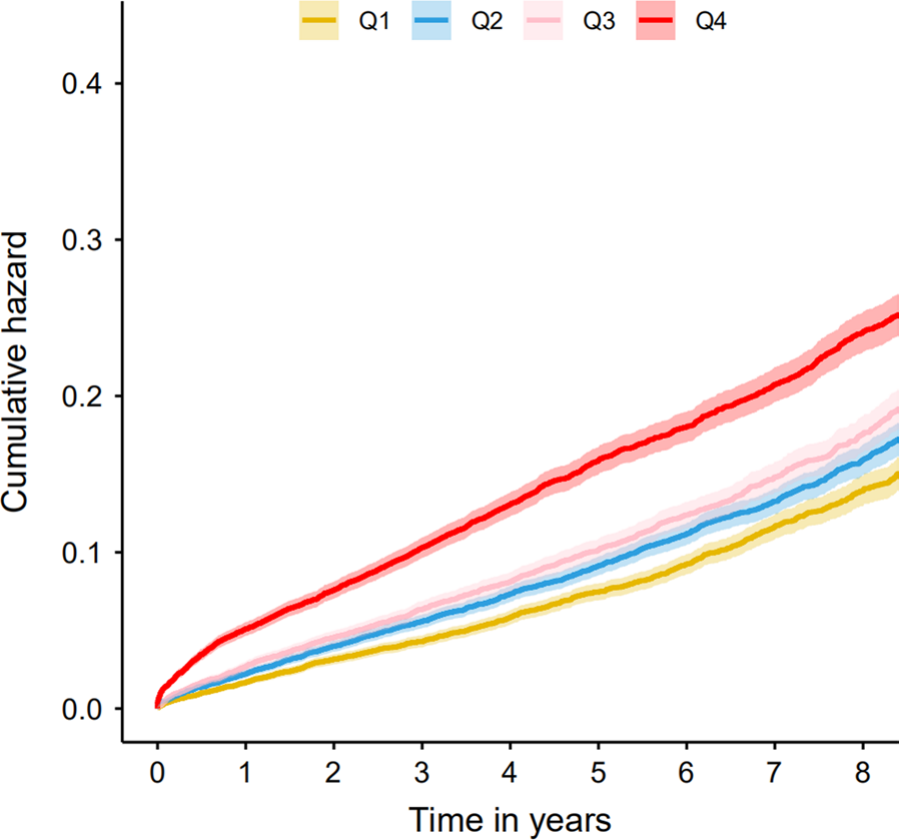
Kaplan–Meier curves for quartile values of plasma levels of PT-INR. Quartile1: PT-INR ≤ 0.96; Quartile2: 0.96 < PT-INR ≤ 1.01; Quartile3: 1.01 < PT-INR ≤ 1.06; Quartile4: PT-INR > 1.06

5,613 (12.57%) patients died in total during the median follow-up period of 5.25 years**.** Our result revealed that patients had significantly higher incidence of long-term mortality with increasing PT-INR level (Fig. 1).

**Fig. 2:**
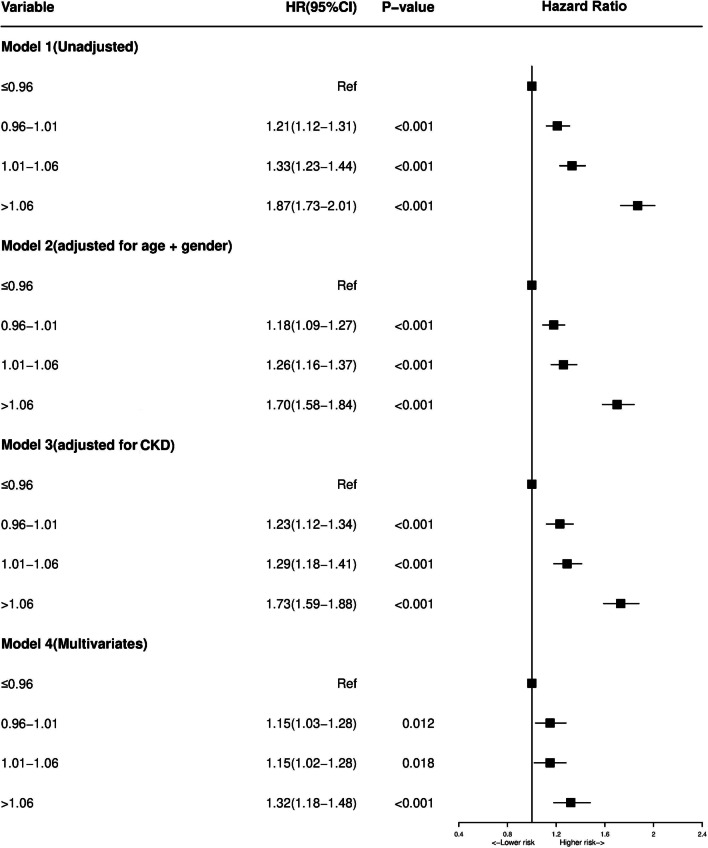
Cox proportional hazard ratios for long-term all-cause mortality in different models. Model 1: Cox proportional hazard ratio for long-term all-cause mortality unadjusted. Model 2: Cox proportional hazard ratio for long-term all-cause mortality adjusted for age > 75 and gender. Model 3: Cox proportional hazard ratio for long-term all-cause mortality adjusted for CKD. Model 4: Cox proportional hazard ratio for long-term all-cause mortality adjusted for multiple variables

**Fig. 3:**
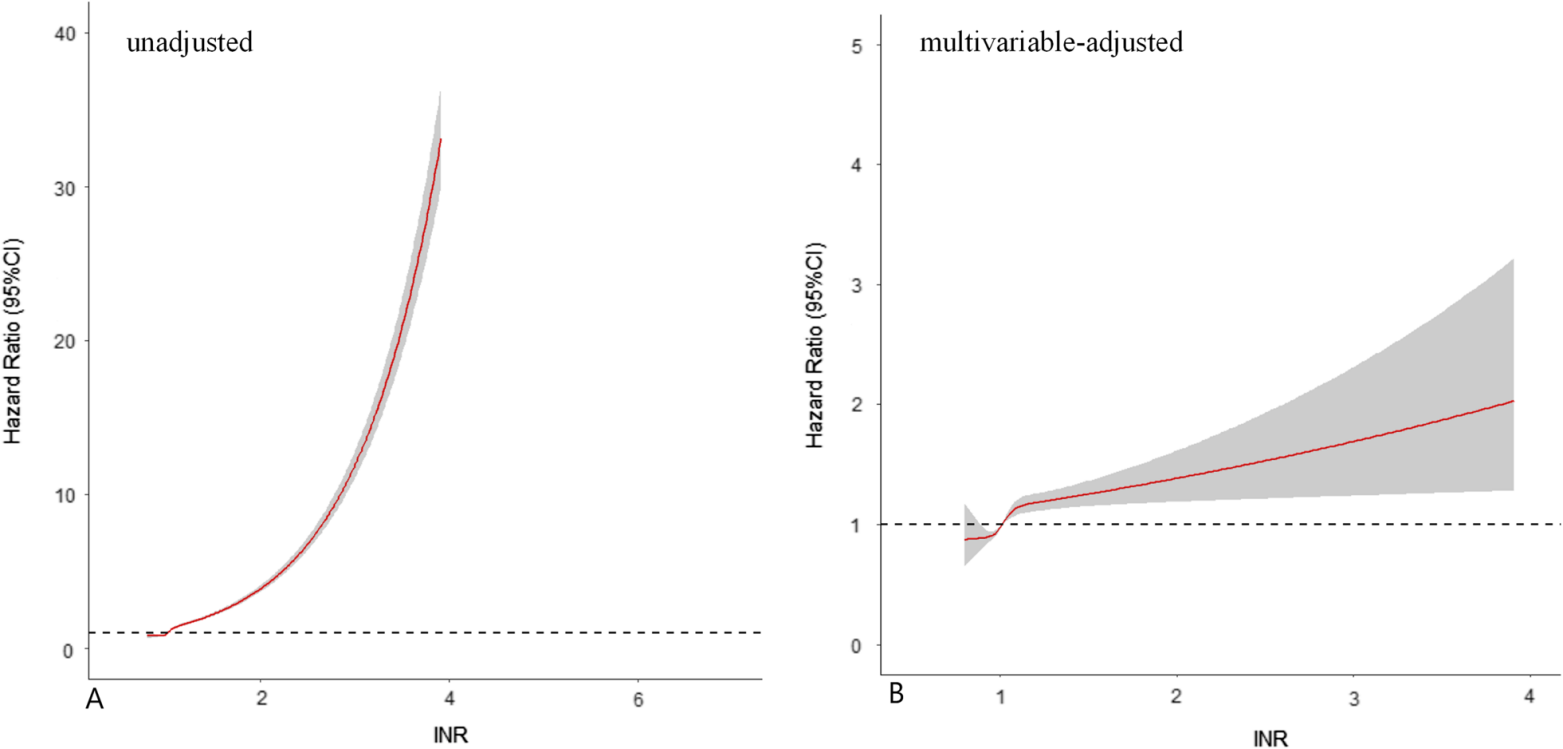
Restricted spline curve of the HbA1c hazard ratio for mortality. **A** The restrict spline curve of univariate cox model. **B** The restrict spline curve of multivariate cox model, adjusted for age, gender, AMI, hypertension, DM, CKD, PCI, CHF, hemoglobin, LDL-C, AST, ALT and FIB

By Univariate regression analysis, we found such as age, DM, CKD and so on were risk factors of the long-term all-cause mortality (Additional file 2: Table S2). Patients in higher PT-INR level developed a significantly higher long-term mortality than other group in multivariate cox models, (Compared with Quartile 1, Quartile 2 [0.96 < PT-INR ≤ 1.01], aHR = 1.00, 95% CI 0.91–1.00, *P* = 0.99; Quartile 3 [1.01 < PT-INR ≤ 1.06], aHR = 1.10, 95% CI 1.01–1.20, *P* = 0.03; Quartile 4 [Quartile4: PT-INR > 1.06], aHR = 1.33, 95% CI 1.22–1.45, *P* < 0.05) (Fig. 2). We observed the risk of all-cause mortality increase with the increase of PT-INR levels in the univariate and multivariate cox models of restricted cubic splines (Fig. 3).

### Sensitivity analysis

**Fig. 4:**
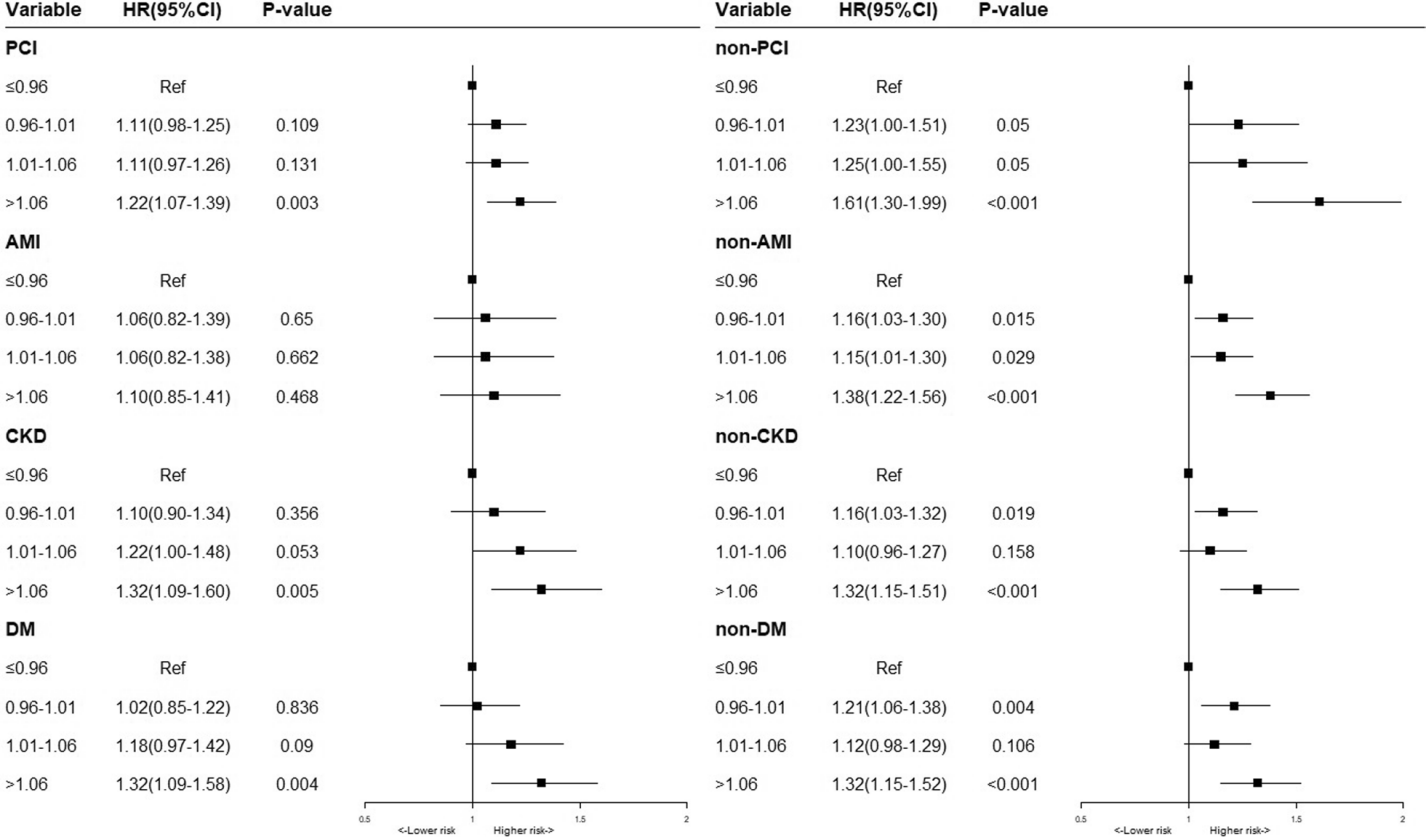
Multivariable cox proportional hazard ratios for long-term all-cause mortality in Subgroups stratified by PCI, AMI, CKD and DM

Sensitive analysis was conducted in different subgroups to explore the relationship between PT-INR levels and all-cause mortality. We observed the stable association between PT-INR levels and long-term mortality in most subgroups, although the risk of higher PT-INR level was reduced slightly in AMI patients (Fig. 4).

## Discussion

To the best of our knowledge, this is the first study to estimate the association of baseline PT-INR level and long-term all-cause mortality in a large cohort of CAD patients without atrial fibrillation or oral anticoagulant therapy. We found that higher PT-INR level, rather than lower PT-INR, is an independent predictor of all-cause mortality in CAD patients.

As a common indicator reflecting coagulation function, PT-INR is widely used in patients with atrial fibrillation treated by warfarin. The optimal PT-INR range of warfarin in the treatment of atrial fibrillation (AF) is about 2–3, which can reduce the occurrence of bleeding or thrombus events [7, 17]. A research found that an adjusted-dose warfarin (PT-INR 2.0–3.0) importantly reduces stroke for high-risk patients in patients with AF and at least one thromboembolic risk factor [18]. The guideline recommend a target PT-INR of 2.0–2.5 for AF patients undergoing percutaneous intervention, because the risk of bleeding will increase sharply when the PT-INR is above the target therapeutic range [19]. A previous study showed that both the increase and decrease of PT-INR will worsen the condition of heart failure patients with non-valvular atrial fibrillation and further increase the risk of death [20]. Tan et al. found that the higher PT-INR (> 1.2) was closely related to the long-term worse prognosis after arterial stripping [10]. Delgado et al. [21] also found that PT-INR INR was an independent risk factor in patients undergoing CAG without oral anticoagulant therapy. While, Delgado et al. focused on caucasians and didn’t excluded patients with AF, which may affected the conclusion by selection bias. An increased body of evidence demonstrate that East Asian population has a lower risk of atherothrombotic event and a higher tendency of serious bleeding during antithrombotic treatment compared with Caucasians [22]. In order to further research, this study was carried out based on Chinese patients in order to figure out the relationship between PT-INR level and long-term mortality in CAD patients without anticoagulant therapy. The result of this study revealed a similar conclusion, a J-shaped relationship between PT- INR and long-term mortality with a larger cohort. In the hypothesis, higher PT-INR levels should be more beneficial to CAD patients, while our study found that patients with a higher PT-INR level had higher risk of long-term mortality compared patients with lower PT-INR levels when complicated CAD without anticoagulation therapy. Many studies revealed that exists a significant variability in the coagulation factor levels between patients affected by acute coronary syndrome (ACS). This variation on coagulation factors levels is due to environmental or genetic determinants [23]. In patients with CAD, especially AMI, the formation of coronary thrombosis can significantly increase the risk of death [24]. Previous study found that a higher occurrence of ischemic complications after ACS may relate to a “hypercoagulable status”, which implied that higher PT-INR level is suitable for ACS patients [25]. Eikelboom et al. enrolled 27,395 patients with stable atherosclerotic vascular disease found that rivaroxaban (2.5 mg twice daily) plus aspirin had better cardiovascular outcomes and more major bleeding events than those assigned to aspirin alone and that rivaroxaban (5 mg twice daily) alone did not result in better cardiovascular outcomes than aspirin alone and resulted in more major bleeding events [26]. This result implied that CAD patients need to keep a balance between the risk of bleeding and coagulation, which consist with our result.

PT-INR is the ratio of a patient's PT to a normal (control) sample and elevated level reflect the risks of bleeding. Bleeding is a common complication in CAD patients, especially in those with ACS [27–29]. Previous study found that plaque hemorrhage is considered as high-risk unstable plaque marker [30], which is commoner among the old patients [31]. Therefore, the lower risk of bleeding may help limit the formation of bleeding within the plaque, which furtherly stabilizes atherosclerotic plaque and reduce the risk of cardiovascular events and death. Moreover, the activation of inflammation and blood coagulation pathways play an important role in the pathogenesis of cardiovascular diseases [32], which can activate each other and affect the prognosis of cardiovascular diseases. Some researches proved that inflammation could prolong PT and increase the risk of bleeding, so higher PT-INR may reflect the active status of inflammation. Meanwhile, the activation of the coagulation system could also accelerate the occurrence of inflammation and lead to a poor prognosis.

Previous studies and clinical practices on CAD patients may focus more on the risk of thrombus load. Our research indicated that higher PT-INR is an independent risk factor of long-term mortality for CAD patients. Previous studies found that dual antiplatelet therapy (DAPT) with aspirin and a P2Y12 inhibitor reduces ischemic recurrences in CAD patients treated with PCI, but increases bleeding events [33, 34]. Our results implied that it is necessary to balance the risk of bleeding and coagulation in these patients in order to improve the prognosis. Further studies are needed to detective the association between PT-INR and bleeding risk in the treatment of CAD patients.

## Limitation

There are several limitations in this study. Firstly, our study included patients from a single center, which may limit the external applicability of the results. However, our cohort is representative because the hospital is the largest cardiovascular center in southern China, involving more than 40,000 patients from southern cities. Secondly, as an observational study, there are uncertain risk factors (such as lifestyle, malignancy) that may cause residual confounding effects on long-term mortality. But the results in our study have adjusted for possible prognostic confounders.

## Conclusion

Our study firstly demonstrates high levels of PT-INR were associated with an increased risk of all-cause mortality. Further studies are needed to detective the association between PT-INR and bleeding risk among CAD patients.

## Supplementary Information

**Additional file 1: Supplementary Table S1.** Univariable Cox regression analysis of long-term all-cause mortality.

**Additional file 2: Supplementary Table S2.** The ICD-10 codes information of diagnoses.

**Additional file 3: Supplemental Figure 1.** The flow of participants through the trial.

## Data Availability

The datasets used and/or analysed during the current study are available from the corresponding author on reasonable request.

## Abbreviations

PT-INR: Prothrombin time-international normalized ratio
CAD: Coronary artery disease
AF: Atrial fibrillation
CAG: Coronary angiography
PCI: Percutaneous coronary intervention
ICD-10: 10Th Revision Codes of the International Classification of Diseases
eGFR: Estimated glomerular filtration rate
MDRD: Modification of diet in renal disease
CKD: Chronic kidney disease
CHF: Congestive heart failure
NYHA: New York Heart Association
DM: Diabetes mellitus
IQR: Interquartile range
ANOVA: Analysis of variance
AMI: Acute myocardial infarction
ACS: Acute coronary syndrome
DAPT: Dual antiplatelet therapy
