# Supplementary material for: The association between prothrombin time-international normalized ratio and long-term mortality in patients with coronary artery disease: a large cohort retrospective study with 44,662 patients
Source: BMC Cardiovasc Disord. 2022 Jun 29;22:297. doi: 10.1186/s12872-022-02619-4 (PMC9245258; doi:10.1186/s12872-022-02619-4)
Supplement: Supplementary file 1 — Additional file 1: Supplementary Table S1. Univariable Cox regression analysis of long-term all-cause mortality. [file 12872_2022_2619_MOESM1_ESM.docx]

**Supplemental Table S1. Univariable Cox regression analysis of long-term all-cause mortality.**

| **variable** | **Univariate** | |
| --- | --- | --- |
|  | HR（95% CI） | p value |
| Age | 1.03(1.03-1.03) | <0.001 |
| Gender | 0.93(0.87-0.99) | 0.017 |
| AMI | 1.16(1.08-1.23) | <0.001 |
| CHF | 2.47(2.29-2.66) | <0.001 |
| HT | 1.19(1.13-1.26) | <0.001 |
| DM | 1.30(1.23-1.38) | <0.001 |
| PCI | 0.90(0.85-0.95) | <0.001 |
| CKD | 2.11(1.99-2.24) | <0.001 |
| INR | 2.04(1.88-2.22) | <0.001 |
| D-dimer | 1.00(1.00-1.00) | <0.001 |
| FIB | 1.15(1.13-1.18) | <0.001 |
| ALT | 1.00(1.00-1.00) | <0.001 |
| AST | 1.00(1.00-1.00) | <0.001 |
| WBC | 1.03(1.03-1.04) | <0.001 |
| HGB | 0.98(0.98-0.99) | <0.001 |
| CHOL | 0.95(0.93-0.97) | <0.001 |
| TRIG | 0.92(0.90-0.95) | <0.001 |
| LDLC | 0.95(0.93-0.98) | 0.001 |
| HDLC | 0.84(0.75-0.93) | 0.001 |
| HbA1c | 1.08(1.05-1.10) | <0.001 |
| URIC | 1.00(1.00-1.00) | <0.001 |
| eGFR | 0.98(0.98-0.99) | <0.001 |
| ALB | 0.93(0.92-0.93) | <0.001 |
| Antiplatelet | 0.83(0.71-0.97) | <0.001 |
| ACEI/ARB | 0.83(0.79-0.88) | <0.001 |
| Beta-blockers | 0.83(0.78-0.89) | <0.001 |
| Statin | 0.69(0.62-0.77) | <0.001 |

**Abbreviation:** AMI: acute myocardial infarction. CHF: Congestive Heart Failure. DM: diabetes mellitus. PCI: percutaneous coronary intervention. CKD: Chronic kidney disease. WBC: white blood cell. HGB: hemoglobin CHOL: Serum total cholesterol.TG: Triglyceride. LDL-C: Low-Density Lipoprotein Cholesterol. HDL-C: Hight-Density Lipoprotein Cholesterol. eGFR: estimated glomerular filtration rate. ACEI/ARB: angiotensin-converting enzyme inhibitor/angiotensin receptor blocker. AST: aspartate transaminase. ALT: alanine aminotransferase. FIB: fibrinogen.
