# Supplementary material for: The association between prothrombin time-international normalized ratio and long-term mortality in patients with coronary artery disease: a large cohort retrospective study with 44,662 patients
Source: BMC Cardiovasc Disord. 2022 Jun 29;22:297. doi: 10.1186/s12872-022-02619-4 (PMC9245258; doi:10.1186/s12872-022-02619-4)
Supplement: Supplementary file 2 — Additional file 2: Supplementary Table S2. The ICD-10 codes information of diagnoses. [file 12872_2022_2619_MOESM2_ESM.docx]

**Supplemental Table S2. The ICD-10 codes information of diagnoses.**

| Diagnosis | ICD-10 codes |
| --- | --- |
| Coronary Artery disease | CD-10; I20.xx–I25.xx, I50.00001 and I91.40001 |
| Hypertension | I10.x00, I10.x05, I10.x04, I10.x03, I11.900, I10. 13, I12.900, H35.004, I12.903, I15.900, I67.400, I13.900, I15.800, I11.000, I12.000, I10.x06, I10.x01, I13.100, I10.x04, I10. 03, I10.x05, I10.x03, R03.000, I11.901, I10.x09, I10.x07, I10.x00, I15.102, I12.900, I11.900, I11.002, I12.000, I10.x11, I13.900, I15.103, I15.200, I11.000, I11.001, I10.x12, I10.x10, I13.000, |
| Diabete mellitus | E11.900, E14.900, E11.901, E11.300, E11.301+H36.0, E11.401+G63.2, E11.501+I79.2, E11.601, E11.700, E11.200+N08.3, E10.900, E13.905, E11.800, E13.903, E11.400, E11.200+N08.3, E13.300+H36.0, E13.200+N08.3, E11.100, E13.907, E11.10001, E11.60001, E14.10001, E11.500, E10.401+G63.2, E11.403+G63.2, E13.400+G63.2, E13.500, E13.700, Z83.300, E11.502, E11.60002, E10.201, E11.901, N08.301*, E11.900, E11.700, E11.200, E11.90002, E11.201+N08.3, E14.900, E13.900, E14.300, E14.800, E13.200, E11.800, O24.300, E14.200, O24.100, E13.800, E11.503, E11.400, E13.600, E14.600, E11.302+H28.0, E14.500, E14.400, E11.500, E11.101, E13.201+N08.3, E11.70001, E14.700, E11.502+I79.2, E13.400, E11.100, E11.103, E11.300, E13.300, E10.400, E13.500, E11.505, E12.000, E11.600, E13.700, E11.604, E11.402+G99.0, R73.003 |
