# Supplementary figures and images for: The association between prothrombin time-international normalized ratio and long-term mortality in patients with coronary artery disease: a large cohort retrospective study with 44,662 patients
Source: BMC Cardiovasc Disord. 2022 Jun 29;22:297. doi: 10.1186/s12872-022-02619-4 (PMC9245258; doi:10.1186/s12872-022-02619-4)

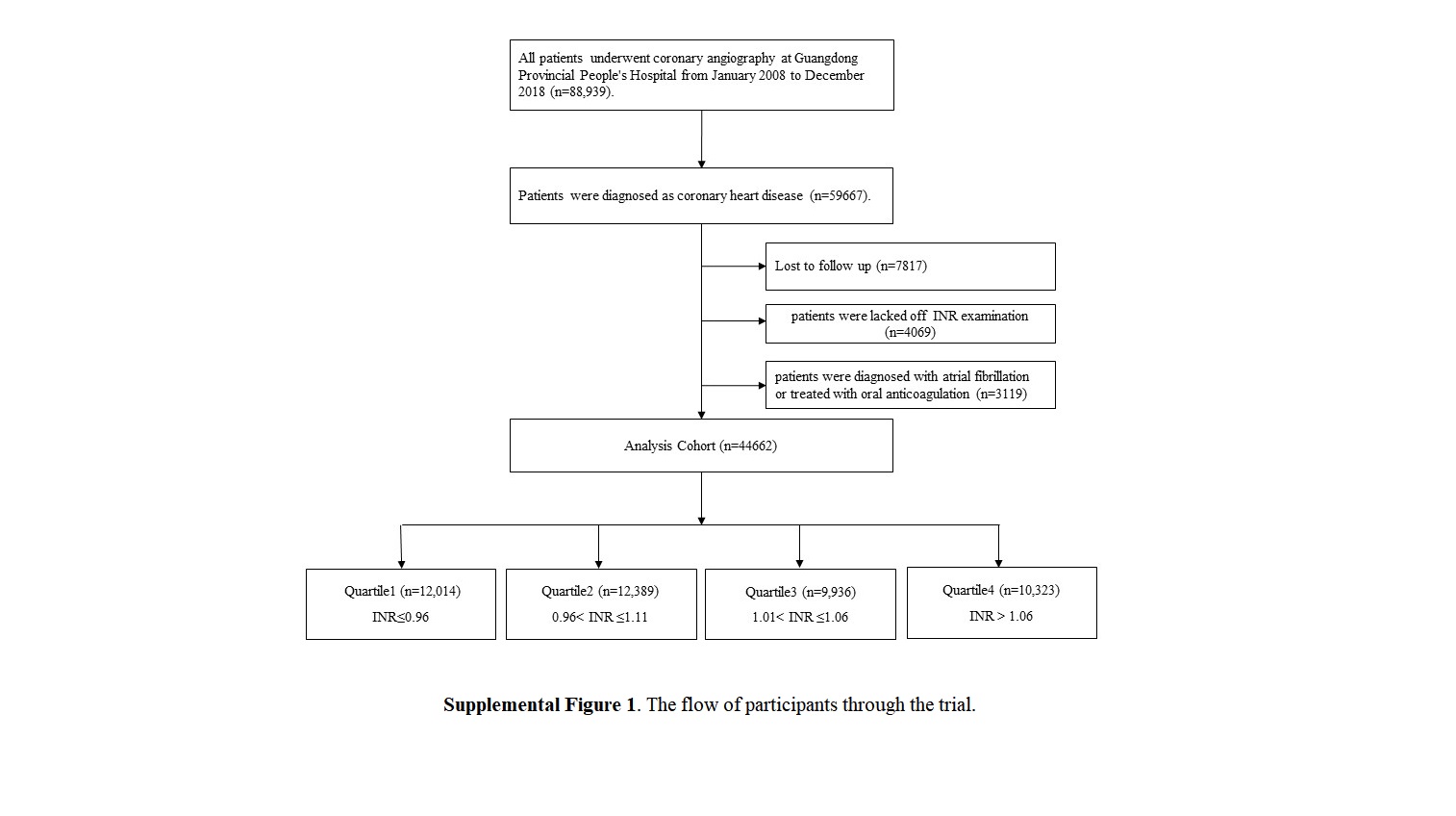

Supplement: Supplementary file 3 — Additional file 3: Supplemental Figure 1. The flow of participants through the trial. [file 12872_2022_2619_MOESM3_ESM.jpg]
